# Decision-Making Under Ambiguity or Risk in Individuals With Alzheimer’s Disease and Mild Cognitive Impairment

**DOI:** 10.3389/fpsyt.2020.00218

**Published:** 2020-03-18

**Authors:** Tingting Sun, Teng Xie, Jing Wang, Long Zhang, Yanghua Tian, Kai Wang, Xin Yu, Huali Wang

**Affiliations:** ^1^Dementia Care and Research Center, Peking University Institute of Mental Health (Sixth Hospital), Beijing Dementia Key Lab, Beijing, China; ^2^National Clinical Research Center for Mental Disorders, Key Laboratory for Mental Health, National Health Commission, Beijing, China; ^3^Department of Neurology, The First Affiliated Hospital of Anhui Medical University, Hefei, China

**Keywords:** decision-making, Alzheimer’s disease, mild cognitive impairment, executive function, memory

## Abstract

**Background:**

Making advantageous decisions is essential in everyday life. Our objective was to assess how patients with mild cognitive impairment (MCI) and Alzheimer’s disease (AD) make decisions under conditions of ambiguity or risk. In addition, the study also aimed to examine the relationship between decision-making competence and memory and executive function.

**Methods:**

Patients with MCI (n = 36) and AD (n = 29) and healthy elderly controls (HC, n = 34) were recruited from the memory clinic. All subjects were administered a comprehensive neuropsychological battery test. We used the Iowa Gambling Task (IGT) to measure decision-making under ambiguity and the Game of Dice Task (GDT) to measure decision-making under risk. Pearson’s correlation was used to examine the relationship between the performance of IGT and GDT with delayed recall and the Stroop test.

**Results:**

In the GDT, MCI and AD patients presented similar performance but showed different patterns when compared with the HC group. The proportion of those making advantageous choices was lower in the AD group than in the HC group (*p* = 0.01), while the MCI and HC groups did not differ (*p* = 0.14). Meanwhile, concerning the ratio of accepting negative feedback, the AD (*p* < 0.01) group was significantly different from the HC patients, but the MCI (*p* = 0.06) and HC groups did not differ. In the IGT, MCI and AD patients selected randomly from advantageous and disadvantageous decks (*p* = 0.94 and *p* = 0.54), showing no significant change in performance over time. In contrast, the HC group made increasingly frequent advantageous selections over time (*p* = 0.04). Furthermore, the proportion of advantageous decision-makers for the GDT had a linear relationship with delayed recall of the Hopkins Verbal Learning Test and Stroop color words (*p* < 0.01 and *p* < 0.01, respectively).

**Conclusion:**

Our findings suggest that decision-making ability under ambiguity is compromised in MCI and AD, and the decision-making under risk is only impaired in AD. Reduced decision-making performance under risk is closely correlated with lower executive functions and memory.

## Introduction

Alzheimer’s disease (AD) is the most common cause of dementia symptoms and involves a progressive decline in many cognitive domains, such as memory, attention, and executive functions (1). Mild cognitive impairment (MCI) is described as a transitional period between normal aging and the diagnosis of clinically probable very early AD, and it involves memory impairment and slight cognitive deficits beyond those expected for age (2, 3).

The impairment of these functions may affect the abilities of patients with AD and MCI, such as decision-making. Several studies have shown that impaired memory, attention, and executive functions most likely compromise decision-making (4–6). Making decisions is essential for these two types of clinical patients in terms of domains of daily life including medical care (choosing between different treatment options), financial issues, and anticipating or planning possible nursing home placement. A study showed that older adults who make fewer advantageous decisions in laboratory decision-making tasks are more vulnerable to being deceived by misleading advertisements than older adults who are good at decision-making (7). In real life and laboratory investigations, there are two general types of decision-making situations: decisions under uncertainty and decisions under risk. Several studies have found that some healthy older adults present difficulties in making advantageous decisions, especially when information about the options is ambiguous, missing, or misleading (8–10). When a situation is complex, people need to learn from experience which options are best for them in the long term (5). The poor ability to learn from experience over time, resulting from compromised executive function (11, 12) and other neuropsychological abilities (5), may account for decision-making difficulties in normal elderly (9). AD and MCI patients, who have more cognitive impairment compared with healthy controls, may have severe difficulties in decision-making under different situations.

Recent research has shown that patients with MCI perform worse than healthy peers in decision-making tasks under risk (13, 14). In this situation, explicit information about the possible results of various options and their associated probabilities is provided, and participants can depend on their own strategy patterns by calculating or estimating (6). Difficulties also emerge in decision-making in situations of ambiguity (14–17). AD patients are also found to show poorer performance than healthy controls in decision-making under risk (8) and ambiguity (15–18), but they do not differ from MCI patients under risk (19) and ambiguity (15–17). However, one study reported a relatively intact decision-making ability of mild AD patients under risk (20). Previous studies have inconsistent results about decision-making in MCI and AD patients. Meanwhile, these studies either studied decision-making just in one group of MCI and AD patients compared with healthy controls or two groups just under one decision-making condition. A recent study provided compelling evidence that low decision-making ability is an early harbinger of adverse cognitive outcomes and a manifestation of accumulating AD pathology in the brain (21). Furthermore, the ability of decision-making in different conditions among individuals with MCI and AD needs further investigation.

Previous studies found the decision-making under risk might be related with executive function (22–24) rather than working memory (22). However, the role of executive function and working memory in decision-making under ambiguity remains unclear (25, 26). There have been controversies on how memory and explicit recall might impact decision-making (27–29). Concerning MCI and AD, previous studies found a positive relationships between executive function and decision making under risk (17, 19). However, Bayard et al. did not observe significant relationship between working memory, executive functions and decision-making under ambiguity (15), while studies by Zamarian and colleagues did in MCI and mild AD (30, 31). Therefore, decision making under risk or ambiguity might have diverse relationship with cognitive functions in MCI and AD. All in all, further studies are needed to understand how neurocognitive functions interact with making advantageous decisions in different situations in AD and MCI patients. It is anticipated the findings would trigger specific strategies that help people with cognitive disorders make a favorable decision in daily life.

To the best of our knowledge, this study is the first to compare the decision-making performance of older adults with MCI and AD in both risky and ambiguous conditions. Because of slighter cognitive deficits in MCI patients than in AD patients, patients with MCI may not show difficulties in decision-making under risk but may perform worse in decision-making under ambiguity. It may be hypothesized that decision-making performance for individuals with MCI under risk is better than that of individuals with AD, but in conditions under ambiguity, the performance of patients with MCI is similar to that of patients with AD. Based on previous decision-making studies, decision-making under risky conditions is measured by the Game of Dice Task [GDT, (22)], and under ambiguous conditions, decision-making is commonly evaluated using the Iowa Gambling Task [IGT, (32)]. In this study, we aimed to investigate the aforementioned hypothesis by comparing the performance of MCI and AD patients with that of healthy controls in two gambling games. Meanwhile, we intended to explore the potential relationship between the decision-making competence and memory and executive function in AD and MCI patients.

## Methods

### Participants

From May to November 2018, 36 patients with MCI and 29 patients with AD were recruited from the case registry of the Dementia Care & Research Center of Peking University Institute of Mental Health. The case registry has been described in a previous study (33). Briefly, the participants completed a standardized neuropsychological assessment, underwent clinical interviews and brain imaging examinations, and received a clinical diagnosis by a memory specialist.

All participants with MCI met Petersen’s MCI criteria as follows: (a) memory problems confirmed by an informant, (b) preserved general cognitive function (minimental state examination (MMSE) score of > 24), (c) intact activities of daily living (an ADL score of ≤ 26), and (d) failure to meet the diagnosis of dementia (34). Other inclusion criteria were as follows: (a) age ≥ 55 years, (b) schooling education (≥5 years), (c) a Clinical Dementia Rating (CDR) score = 0.5 and (d) a Hamilton Depression Scale (HAMD) score of < 12. The exclusion criteria of MCI were as follows: Axis I psychiatric disorders listed in the Diagnostic and Statistical Manual of Mental Disorders 4th edition (DSM-IV); history of stroke, subdural hematoma, tumor, other intracranial space-occupying diseases or cerebrovascular disorders, and presence of significant risk factors for cerebrovascular disorders (i.e., a score higher than 4 on the modified Hachinski Ischemia Scale); current or previous neuropsychiatric diseases such as Parkinson’s disease, epilepsy; and presence of a physical illness that could affect cognition.

A clinical diagnosis of AD was made according to the criteria for dementia cited in the International Classification of Diseases, 10th Revision (34). Other inclusion criteria were as follows: more than 6 months’ duration of the disease and an MMSE score of 15–24 [for more details, see (35)].

Thirty-four participants met the inclusion criteria of healthy controls. They underwent the neuropsychological assessments and CDR to exclude cognitive impairment. Healthy controls met criteria as follows: (a) age ≥ 55 years; (b) with more than 5 years of schooling education; (c) with preserved general cognitive function [MMSE score of > 24 and Montreal Cognitive Assessment (MoCA) score of >26]; (d) a CDR score = 0; and (e) a HAMD score of < 12.

The present study was approved by the Ethics Committee of Peking University Institute of Mental Health (Sixth Hospital), Beijing, China. All participants were fully informed regarding the study protocol and provided written informed consent.

### Neuropsychological Tests

All participants underwent a neuropsychological assessment. For the purpose of this study, we included the score of the MMSE, the MoCA, the Hopkins Verbal Learning Test (HVLT) and the Stroop color word tasks.

### Decision-Making Under Risk

The GDT is often used to measure decision-making under risk conditions (22). Participants are asked to sit in front of a computer screen and watch the computer throwing dice and to choose among different alternatives that are explicitly related to a specific amount of gain/loss and that have distinct winning probabilities. Before each throw, they can choose a single number or a combination of two, three, or four numbers. If one of the numbers of the combination that they choose is thrown with the die, the participants receive the associated amount of money. In contrast, the subjects lose the same amount of money when none of the chosen numbers is thrown. In this task, subjects are asked to maximize the starting fund (1,000 *yuan*) within 18 throws. One single number with a winning probability of 1/6 and a combination of two numbers with a winning probability of 2/6 are defined as disadvantageous or risky decisions, and a combination of three numbers and four numbers are defined as advantageous or nonrisky decisions. Selecting advantageous options leads to a positive outcome throughout the test, whereas selecting disadvantageous options leads to a negative outcome [for more details about the rules of GDT, see (36)].

We calculated the (a) final capital and (b) net score (the number of nonrisky options minus the number of risky options) and (c) utilization of negative feedback. If participants chose a disadvantageous option (one number or the combination of two numbers) and obtained a loss and then in the next trial immediately chose an advantageous choice, we identified this behavior as “using negative feedback.” In contrast, if participants chose a disadvantageous option immediately after receiving a loss for a disadvantageous option, we defined this behavior as “not using negative feedback.” The utilization of negative feedback is the frequency of choosing advantageous option after choosing a disadvantageous option divided by the frequency of using negative feedback; and (d) the frequency of choosing each of the four possible choices, making disadvantageous choices, and making advantageous choices.

### Decision-Making Under Ambiguity

The IGT is used to measure decision-making under conditions of uncertainty (37). Participants must choose between four different decks (A, B, C, D). Card selections from decks A and B result in large monetary gains followed by large penalties at unpredictable times, leading to a negative balance. Therefore, we define decks A and B as disadvantageous choices. Decks C and D are advantageous choices because they lead to moderate gains but also to moderate or low losses, leading to a positive final balance. Participants attempt to solve the task successfully but are not told the rules for gains and losses [for more details about the rules of IGT, see (38)].

We calculated the following: (a) the net score, which was selected from decks C and D minus selections from decks A and B. One hundred choices were equally divided into five blocks. The calculation of the net score for each block was used to quantify the progressive change in the selection across the IGT; (b) the utilization of negative feedback: if participants chose a disadvantageous option (A and B) and obtained a loss and then, in the next trial, immediately chose an advantageous choice (C and D), we identified this behavior as “using negative feedback.” If the opposite occurred, we defined the behavior as “not using negative feedback”; (c) the frequency of making four possible choices; (d) the frequency of advantageous and disadvantageous choices; (e) the advantageous profile: a positive net score [(C+D) − (A+B) > 0] indicates more frequent selection from advantageous decks, whereas a negative net score [(C+D) − (A+B) < 0] indicates more frequent selection from disadvantageous decks. We designed five blocks into two parts: the initial phase (trials 1–40; Blocks 1 and 2) and the second part of the IGT (trials 41–100; Blocks 3, 4, and 5). We defined the number of subjects whose net score on the second part was positive as an advantageous profile; and (f) the change ratio in frequency of choosing advantageous choices among Blocks 1–5; that is, the result is the frequency of making advantageous choices in Block 1 subtracted from the frequency of making advantageous choices in each block, divided by the frequency of making advantageous choices in Block 1. For example, the result is the subtraction of the frequency of making advantageous choices in Block 1 from the frequency of making advantageous choices in Block 2, divided by the frequency of making advantageous choices in Block 1 ((B2-B1)/B1).

### Statistical Analysis

All statistical analyses were conducted using the Statistical Package for the Social Sciences (SPSS) version 20.0 for Windows. The data were examined for normal distribution (tested with the Kolmogorov-Smirnov test) and homogeneity of variance (tested with the Levene test). The variables were normally distributed (*p* > 0.05).

Because the neuropsychological tests were associated with age and education, the neuropsychological testing data of the three groups were compared with these two demographic variables as covariates. An analysis of covariance (ANCOVA) with age and education as covariates and with the group as the between-subjects factor was performed to examine the measures of GDT and IGT. For the measures of IGT, we used an ANCOVA with age and education as covariates and with the group as the between-subjects factor for the variables (for all 100 trials) of IGT. For the score of the blocks, we conducted a repeated measures analysis of variance (ANOVA) with age and education as covariates and with block (1–5) as the within-subject factor and group (AD, MCI, controls) as the between-subjects factor for the frequency of advantageous choices and a repeated-measures ANOVA with age and education as covariates and for the advantageous choices of each group (AD, MCI, controls). Finally, relationships between the neuropsychological tests and performance on the gambling tasks were determined using Pearson product-moment correlations.

## Results

### Demographic Characteristics

The AD and MCI participants were older (both *p* < 0.01) than the controls, but the AD and MCI participants did not differ in age. The AD group was less educated than the MCI group (*p* < 0.01) and the controls (*p* < 0.01), with no difference between the controls and the MCI participants. The groups were matched for sex (see **Table 1**).

**Table 1:** Demographic and cognitive performance in three groups.

	AD (N = 29)	MCI (N = 36)	Control (N = 34)	F/χ2	*p*
Age	75.1 (7.92)*	76.9 (7.27)*	65.1 (6.82)	25.86	<0.001
Sex (men/women)	11/19	11/25	15/19	1.38	0.502
Education	11.5 (3.57)*	13.8 (3.03)	13.6 (2.79)	5.26	0.007
MMSE	20.0 (4.76)*^#^	26.6 (2.41)*	29.3 (2.54)	41.97	<0.001
MoCA	13.8 (4.70)*^#^	22.3 (3.38)*	26.6 (2.02)	72.33	<0.001
StroopCW	18.8 (8.77)*^#^	29.3 (10.83)*	38.5 (9.81)	15.11	<0.001

MMSE, minimental state examination; MoCA, Montreal Cognitive Assessment, HVLT-DR, Hopkins Verbal Learning Test Delayed Recall; StroopCW, Stroop color word tasks.

*vs. controls p < 0.05, ^#^MCI vs. AD p < 0.05.

### Neuropsychological Tests

The AD participants performed worse than the MCI participants (*p* < 0.001) and controls (*p* < 0.001) on the MMSE. A significant difference was also observed between the MCI participants and controls (*p* = 0.02). The same situation was also found for MoCA in that the AD participants performed worse than the MCI participants (*p* < 0.001) and controls (*p* < 0.001), and the MCI participants and controls (*p* < 0.001) were found to be significantly different.

Poorer executive functions were found with the Stroop color word test (MCI vs. controls, *p* < 0.001; AD vs. controls, *p* < 0.001). Additionally, the AD group performed worse than the MCI group (all *p* < 0.001). With regard to the performance of memory function on the HVLT Delayed Recall (HVLT-DR), the AD (*p* < 0.001) and MCI (*p* < 0.001) groups were significantly different from the controls (see **Table 1**).

### Decision-Making Under Risk

When controlling for age and education, between-group differences were observed for the final capital (*p* < 0.001). The AD and MCI groups were significantly lower than the controls (*p* < 0.001 and *p* < 0.01, respectively), with no difference between the AD and MCI groups (*p* = 0.10) in the final capital.

An ANCOVA with age and education as covariates and with group as the between-subjects factor was performed to examine the utilization of negative feedback (*F* = 5.08, *p* = 0.008, η^2^ = 0.10). The AD patients showed a lower utilization of negative feedback than the controls (*p* < 0.01), but the MCI group did not differ from the controls (*p* = 0.14).

**Figure 1:**
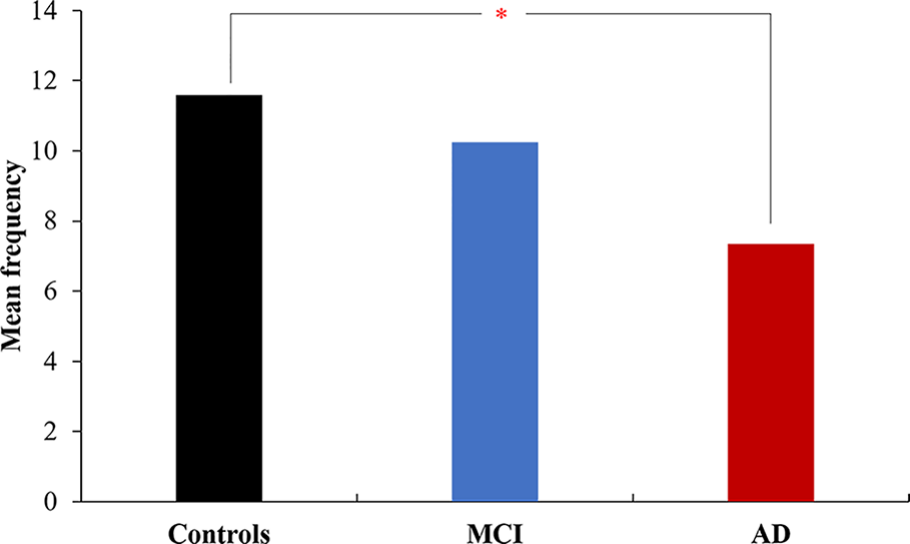
The mean frequency of advantageous decisions in the three groups. The AD patients made fewer advantageous choices than the controls. The MCI patients did not differ from the controls. *p < 0.05.

**Figure 2:**
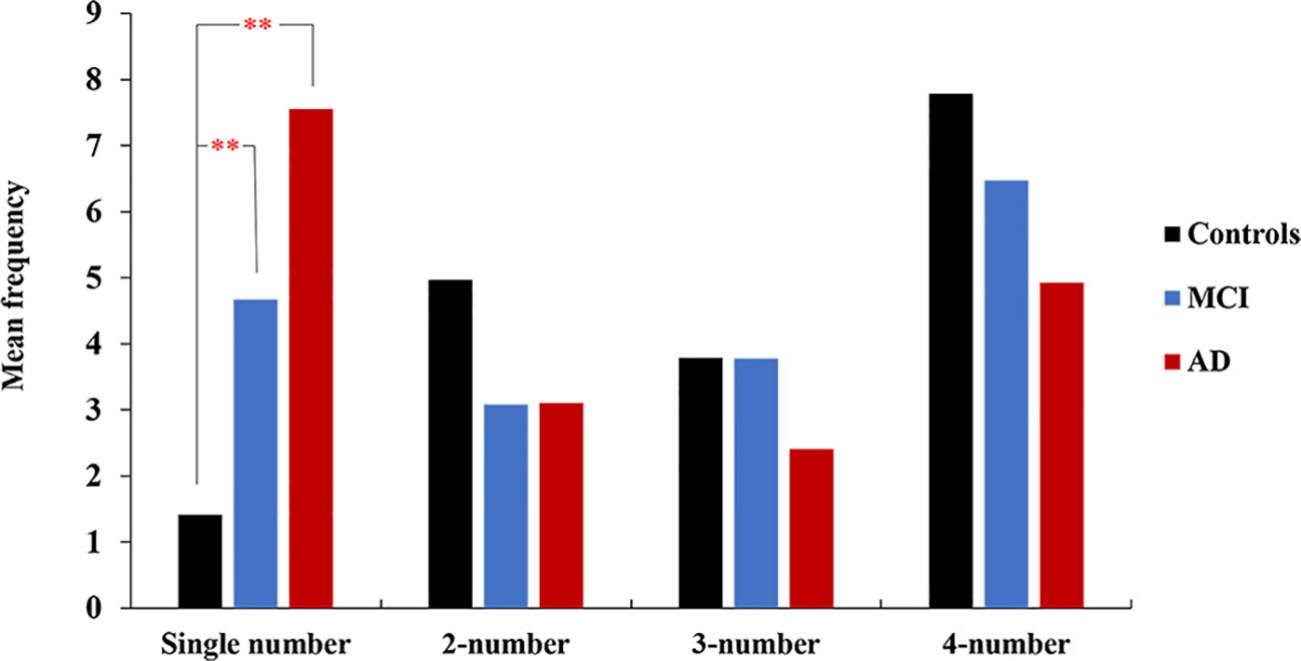
The AD and MCI patients selected one number most frequently, and the controls were more likely to select four-number combinations. Single comparisons between groups revealed significant differences in the frequency of choosing one single number but not two numbers, three numbers and four numbers. **p < 0.01.

When controlling for age and education, between-group differences were observed for the frequency of advantageous choices (*p* = 0.04) and the frequency of single numbers (the most disadvantageous choice) (*p* < 0.001). The AD patients showed a lower preference for advantageous options than the controls (*p* = 0.01), but individuals with MCI showed no difference from the AD group or controls (**Figure 1**). In addition, individuals with AD (*p* < 0.01) and MCI (*p* < 0.001) selected more single number options than the controls (**Figure 2**). For the other three options, there was no difference among the three groups (see **Table 2**).

**Table 2:** The comparison of the performance on the Game of Dice Test (GDT) in three groups [mean (SD)].

	AD (N = 29)	MCI (N = 36)	Control (N = 34)	F/χ2	*p*
Net score	−3.3 (9.11)*	2.5 (9.45)	5.2 (13.65)	3.43	<0.05
Final capital	−4,113.8 (3,342.45)**	2,094.4 (3,465.25)**	23.5 (2,726.64)	9.74	<0.001
Feedback (%)	0.4 (0.25)*	0.6 (0.28)	0.7 (0.38)	5.08	<0.01
Single number	7.6 (4.96)**	4.7 (4.22)**	1.4 (3.52)	10.53	<0.01
2-number	3.1 (2.41)	3.1 (2.22)	5 (5.91)	0.89	0.414
3-number	2.4 (1.8)	3.8 (2.72)	3.8 (4.56)	0.67	0.515
4-number	4.9 (4.1)	6.5 (5.20)	7.8 (7.2)	1.95	0.148
Advantageous choices, n (%)	10.7 (60)*	7.8 (40)	6.4 (40)	3.43	<0.05
Disadvantageous choices, n (%)	7.3 (40)*	10.3 (60)	11.6 (60)	3.43	<0.05
A/D ratio	1.4 (2.08)**	2.5 (3.40)*	4.7 (6.02)	5.38	<0.01

A/D, Frequency of making advantageous choices/Frequency of making disadvantageous choices.

*vs. controls p < 0.05, **vs. controls p < 0.05.

### Decision-Making Under Ambiguity

Based on the adjusted ANCOVA analysis for the IGT total net score, the three groups did not differ. A repeated-measures ANOVA, with block as the within-subject factor and group (AD, MCI, controls) as the between-subjects factor was conducted on the frequency of four choices (A, B, C, D). The results showed that there was no significant effect of block and group on the frequency of three choices (A, C, D). The interactions between block and group on the frequency of choice A (*p* = 0.01) and choice D (*p* = 0.02) were significant. For choice B, the main effect of group (*p* = 0.02) as well as the interaction between block and group (*p* = 0.01) were significant. Overall, the AD patients showed significant differences compared to the healthy controls (*post hoc* contrasts *p* < 0.001), whereas the MCI patients and controls did not differ from each other in the frequency of choice B.

A repeated-measures ANOVA (adjusted with age and education) with block (Blocks 1–5) as the within-subject factor and group (AD, MCI, and controls) as the between-subjects factor was conducted on the change ratio of the frequency about advantageous choices. The results showed that there was a significant effect group on the change ratio in frequency of advantageous choices (*p* = 0.03), and AD patients showed significant differences from MCI patients (*post hoc* contrasts *p* < 0.01). The interaction between block and group on advantageous choices (*p* < 0.01) was significant.

**Figure 3:**
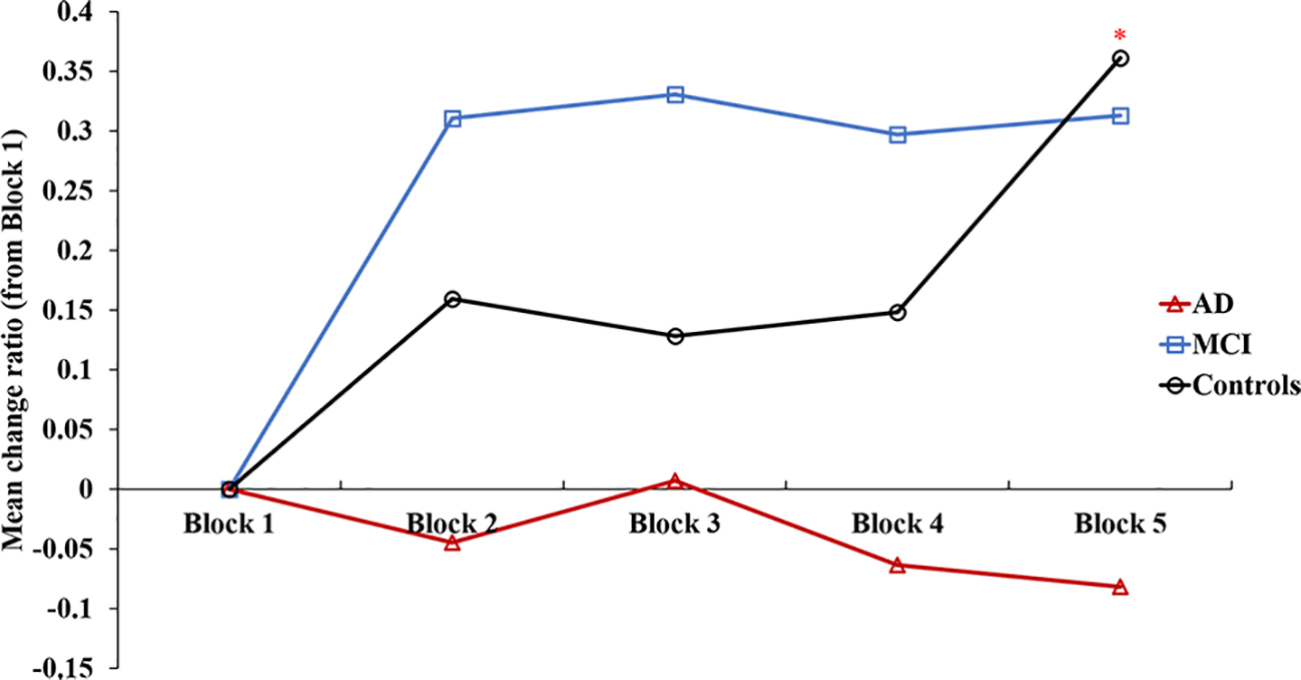
The change ratio of making advantage choices in an Iowa gambling task for 1-5 blocks. Controls selected more advantageous choices over time, whereas the MCI did not differ, and AD chose advantageous choices more randomly over the task. *vs Block 2, Block 3, Block 4 *p* < 0.05.

A repeated-measures ANOVA for the change ratio among Blocks 1–5 of each group (AD, MCI, and controls), adjusted with age and education, found that the controls selected more advantageous choices over time (block effect *p* = 0.04), whereas the MCI (*p* = 0.94) did not differ and AD (*p* = 0.54) made advantageous choices more randomly over the task (**Figure 3**).

Based on an adjusted ANCOVA analysis for the frequency of advantageous choices and the ratio of advantageous choices to disadvantageous choices, there was no difference among the three groups. A statistically nonsignificant trend was observed for the advantageous profile.

### Correlations Between Decision-Making Competence and Cognitive Performance

The GDT variables, including final capital, net score, utilization of negative feedback, frequency of a single-number choice and frequency of advantageous choices, correlated significantly with neuropsychological performance (Stroop color word test, HVLT-DR) (see **Table 3**). However, when education was adjusted, there was no significant correlation between the GDT variables and neuropsychological performance (all *p* > 0.05). We did not observe a significant correlation between the IGT variables and neuropsychological performance.

**Table 3:** Correlations of the performance of gambling dice test with the executive function and memory test.

	Final capital	Net score	Utilization of negative feedback	Single number	Advantageous choices	A/D ratio
Age	−0.152	−0.052	−0.041	0.253^*^	0.052	0.047
Education	0.321^**^	0.277^**^	0.247^*^	−0.312^**^	−0.277^**^	0.287^**^
StroopCW	0.418^**^	0.296^**^	0.346^**^	−0.477^**^	−0.296^**^	0.168
HVLT-DR	0.437^**^	0.358^**^	0.355^**^	−0.555^**^	−0.358^**^	0.252^*^

A/D, Frequency of making advantageous choices/Frequency of making disadvantageous choices; StroopCW, Stroop color word tasks; HVLT-DR, Hopkins Verbal Learning Test Delayed Recall. *p <0.05, ** p <0.01

## Discussion

The present study investigated decision-making in patients with AD and MCI in two gambling tasks under risk or ambiguity. We assessed many measures of the GDT and IGT, as stated in the methods. In the GDT, the AD patients utilized less negative feedback and chose more disadvantageous options than the healthy controls. This finding indicates that AD patients learn very little from information over time and prefer to choose unfavorable options. However, the MCI patients did not differ from the healthy controls. In the IGT, with regard to performance changes over the task, the healthy controls had a stronger tendency toward safe and advantageous responses than the AD and MCI patients. While the healthy controls demonstrated learning as the task proceeded, the AD and MCI patients did not adapt their strategies. In this task, the profile of decision-making for the MCI patients resembled that of the AD patients.

In the GDT, the AD patients chose more risky options than the healthy controls. This result is inconsistent with the finding by Delazer et al. that patients with mild AD chose safe alternatives as frequently as healthy elderly persons (20). It might be partly due to the heterogeneity of the illness. An earlier study found that subjects with worse emotional control abilities chose more risky options (23). People with AD may present impairment in emotional control (23, 39) and executive function (5) in addition to memory decline. However, the results of this study that people with AD chose more risky options than healthy controls are in line with previous investigations (31, 40). Recent investigations have credited the important role of executive functions and numerical processing in decision-making under risk (5, 6, 22, 36, 41), as the prefrontal cortex is involved in both processes (5, 42). Additionally, executive function and numerical training improved their performance in decision-making under risk (43). Therefore, the presence of executive function and numerical processing impairments in AD patients may be the main cause of poor performance in the GDT.

The MCI patients showed no significant difference from the controls and AD patients. This result is inconsistent with previous studies of decision-making under risk in patients with MCI. In two previous studies, MCI patients performed worse than healthy controls (14, 19). Compared with these two previous studies, although this study examined the decision-making ability of MCI patients under risk, this study used different gambling games. The GDT used in this study may not be as sensitive as the tasks in previous studies, or the MCI of patients in this study may have been more severe than in previous studies. These two reasons may be the main cause of this situation.

In the IGT, the AD and MCI patients showed significant differences from the healthy controls. The AD and MCI patients made random decisions and showed poor strategy stability. In contrast to the two groups of clinical patients, the healthy controls made increasingly frequent advantageous selections over time. This finding suggests that the healthy controls assessed the advantageous decks more favorably than the disadvantageous decks and learned to decide advantageously by utilizing feedback and modifying their strategy over time, but the patients with AD and MCI did not. The response patterns of the two groups of clinical patients may be attributed to deficits in memory and executive function, which prevents them from establishing new stimulus-reward relationships and eliminating previously learned responses due to the parietal and temporal atrophy they present (44). Another possible explanation of these results is a dysfunctional ventromedial prefrontal cortex (VMPC) in patients with AD (45–47) and MCI (48). The VMPC is supposed to mediate the use of feedback for current decisions (49). The somatic marker hypothesis was based on the defective decision-making about VMPC damage, which suggested that decision-making is often assisted by emotional processes and somatic “markers” (49, 50). However, little study about the relationship between decision-making damage and VMPC in AD and MCI patients was found. In the future, more confirmatory studies are needed to eliminate even the most resilient skepticism in this regard.

These results verify earlier reports that in MCI patients, the performance of decision-making under ambiguity mimicked that of AD patients and was impaired compared with that of healthy controls (14, 15, 17). The MCI patients manifest slight impairments in cognitive functions, which do not meet the criteria for a diagnosis of dementia (2, 3), and less VPMC atrophy than AD patients (51). In addition, a study found that lower executive functions are required to make advantageous decisions in situations of risk than in situations of ambiguity (19). Therefore, the lower cognitive impairments and relatively intact VMPC function of MCI patients in comparison with those of AD patients may be the reason for this study’s finding that persons with MCI show no difficulties in making advantageous decisions under risk but have difficulties in situations of ambiguity. We can infer that MCI patients may still have intact competence when making decisions under conditions of risk but show impairment in decision-making under ambiguity.

The observation that individuals with MCI and AD did not differ in IGT is similar to previous studies (15, 17, 52). It may indicate that people with MCI and AD have similar disadvantageous decision-making profile in the IGT. However, in our study the MCI subjects exhibited more positive changes from baseline up to the Block 4 task in IGT test compared with control group. It may imply that MCI individuals preserve part neuroplasticity in learning. With repeated trials, individuals with MCI might learn from the feedback over time and make more advantageous choices. Further investigations are warranted to explore the potential mechanism.

In this study, we implemented a correlation analysis to explore the possible contribution of memory and executive functions to decision-making performance. The results indicated that the capacity to utilize feedback in the decision-making under risk was associated with good executive ability and good memory. Executive functions contribute to decision-making under risk by guiding the categorization of information and alternatives, the development and application of strategies and the integration of feedback (5, 19). People usually depend on declarative memory to form and update a long-term representation that integrates the variations in reward and punishments across decks and across experiences during the process of decision-making (29). Without such relational record, an individual has no choice but to rely on the immediately available information and thus, the decision sticking to a certain deck or switching to another deck relies on each single outcome (29). Therefore, intact episodic memory is important for making good decisions (14). The memory deficit in conditions, such as AD and MCI, could trigger the impairment on decision-making (28, 29).

We identify two possible limitations of our study. First, the experimental methods we used may not reflect the actual deficits in decision-making. A study showed that older adults who make fewer advantageous decisions in laboratory decision-making tasks are more vulnerable to being deceived by misleading advertisements (7). Therefore, it would be interesting to validate our findings with real-world decision-making tasks. Second, we observed the great variation in performance on IGT and GDT in three groups. It might be partly attributed to not only the sample size but also the potential neuropathological heterogeneity of the subjects.

In conclusion, to our knowledge, we present the first study that shows that individuals with MCI do not make exactly the same decisions as individuals with AD under conditions of ambiguity and risk. This study finds that AD patients have difficulty making advantageous decisions under ambiguity and risk; however, MCI patients have problems making advantageous decisions under ambiguity but not under risk. We also document the relationship between the decision-making measures under risk and cognitive performance. The capacity tested by GDT and IGT may be considered as the analogue of real-world decision-making, which is essential for care planning and financial arrangement in one’s daily living (20, 53, 54). Therefore, our study highlights the significance of measuring the decision making under ambiguity for early detection of MCI. In the future, more real-life decision-making needs to be performed in patients with MCI and AD, and more longitudinal studies should be conducted to verify that low decision-making ability is associated with increased risk for incident AD and MCI.

## Data Availability Statement

The datasets generated and analyzed during the current study are not publicly available because we are preparing an additional manuscript. However, they are available upon reasonable request to the corresponding authors, TX (xieteng@gmail.com) and HW (huali_wang@bjmu.edu.cn).

## Ethics Statement

This study was carried out in accordance with the recommendations of the Institutional Review Board of Peking University Sixth Hospital with written informed consent from all subjects. All subjects gave written informed consent in accordance with the Declaration of Helsinki. The protocol was approved by the Institutional Review Board of Peking University Sixth Hospital.

## Author Contributions

TS, TX, JW, XY, and HW conceived the study and supervised the work. TS, JW, and HW designed the study and collected the data. TS, TX, LZ, YT, and KW analyzed the data. TS and TX wrote the manuscript. All authors contributed to the subsequent drafts and approved the final version.

## Funding

This work was supported by the National Research and Development Grant from the Ministry of Science and Technology (2017YFC1311100, 2018YFC1314200) and Beijing Municipal Science and Technology Commission (Z161100000516001).

## Conflict of Interest

The authors declare that the research was conducted in the absence of any commercial or financial relationships that could be construed as a potential conflict of interest.
